# Sex-Related Differences Between Patients Undergoing Surgery for Acute Type A Aortic Dissection

**DOI:** 10.7759/cureus.60276

**Published:** 2024-05-14

**Authors:** Baku Takahashi, Keiji Kamohara, Hiroyuki Morokuma, Junji Yunoki

**Affiliations:** 1 Department of Thoracic and Cardiovascular Surgery, Faculty of Medicine, Saga University, Saga, JPN

**Keywords:** aortic surgery risk factors, cardiovascular surgical interventions, sex differences, acute type-a aortic dissection, sex

## Abstract

Introduction

This study aimed to evaluate the sex-specific characteristics and surgical outcomes in patients with acute type A aortic dissection (ATAAD).

Materials and methods

We reviewed the surgical records of patients who underwent ATAAD repair at our institution between 2004 and 2020 (n=213).

Results

Of the 213 patients, 100 (46.9%) were male, and 113 (53.1%) were female. Males were younger than females (62.5 vs. 72.9 years, p<0.0001). Females had more nonspecific symptoms (p=0.04), more frequently developed ATAAD before noon (45.0% vs. 53.1%, p=0.01), and had a significantly longer time from onset to surgery (425.1 vs. 595.8 min, p=0.03). The ascending aorta was replaced more frequently in females than in males (54.5% vs. 72.8%, p<0.01). No significant difference was observed in the in-hospital mortality rate between males and females (9.0% vs. 10.6%, p=0.69). The multivariable logistic analysis demonstrated that being male was not an independent predictor of operative mortality (OR, 0.96; 95% CI, 0.18-5.21; p=0.96). At 10 years, males had significantly better long-term survival rates in the unadjusted cohort than females (79.4% vs. 55.9%, p=0.02).

Conclusions

Male sex was not an independent predictor of early death in patients with ATAAD after surgery, although significant differences were noted in terms of age, onset time, chief complaint, imaging findings, and surgical procedures. A sex-based management strategy involving specific differences should be considered to improve outcomes.

## Introduction

The effects of sex differences on surgical outcomes have been widely deliberated. In the field of cardiac surgery, female sex is an independent risk factor for mortality in coronary artery bypass surgery [1,2] and mitral valve surgery [3,4]. Acute type A aortic dissection (ATAAD) is a life-threatening thoracic cardiac condition. Studies investigating sex differences in ATAAD have reported various findings, with many indicating no significant disparity in mortality rates between males and females and none identifying sex as a risk factor for mortality [5-9]. Some recent reports have indicated that the mortality rate is higher for males [10], whereas others have indicated that females have higher rates [11]; thus, no definitive conclusions have been reached yet.

Considering that the mortality rate of ATAAD increases by 1%-2% per hour, up to two days after onset [12], understanding the sex-specific differences during the period from symptom onset to surgery and the influencing initial symptoms becomes imperative for diagnosis. Furthermore, it would be beneficial for surgeons to understand the differences in the type, extension of the dissection, and location of the primary entry because the surgical procedures can vary depending on these findings [13-15]. However, few studies have delved into these aspects, and previous studies were limited by shorter follow-up periods. Thus, this study aimed to clarify the sex-specific differences in preoperative patient characteristics and both short- and long-term outcomes of ATAAD at our institution, making it the first study to report the differences between males and females in the period from onset to surgery and the location of primary entry in ATAAD.

## Materials and methods

Study design and participant selection

In this retrospective study, we enrolled patients with ATAAD who underwent surgery at Saga University Hospital (Saga, Japan) between January 2004 and December 2020. One patient diagnosed with iatrogenic ATAAD was excluded. All data were collected from medical records and telephone surveys.

Surgery

Our surgical strategy for ATAAD has been described [16]. All operations were performed using a median sternotomy with standard cardiopulmonary bypass (CPB). The attending surgeons determined the cannulation sites and numbers. Brain protection was performed with antegrade and/or retrograde cerebral perfusion under hypothermic conditions, at the discretion of the attending surgeons. The primary entry was mostly resected, and the extent of the aortic surgery was individualized based on patient-specific considerations.

Statistical analysis

The JMP software (version 16.0; SAS Institute, Cary, NC, USA) was used for all statistical analyses. Differences between groups for continuous data were expressed as mean ± standard deviation using unpaired student’s t-test. Categorical variables were expressed as frequencies and percentages using the χ^2^ test or Fisher’s exact test.

A Kaplan-Meier survival curve and log-rank test assessed the rate of freedom from death. Binomial logistic regression analysis was performed to determine the independent predictors of in-hospital mortality. The variables used for the multivariate model were the clinical variables listed in Tables 1, 2, in addition to male and female sex, which were identified using a forward stepwise approach with a cut-off p-value for inclusion and exclusion of 0.1. p-values <0.05 were considered significant for all analyses.

**Table 1:** Preoperative data Values are n (%) or mean ± standard deviation. AF, atrial fibrillation; CAD, coronary artery disease; CVD, cerebrovascular disease; AR, aortic valve regurgitation; IMH, intramural hematoma; RCC, right common carotid artery; RC, right clavicular artery; LCC, left common carotid artery; LC, left clavicular artery.

	Total cohort (n=213)		
Admission variables	Males	Females	p-value
Number of patients (%)	100 (46.9)	113 (53.1)	
Patient age (y)	62.5 ± 13.4	72.9 ± 10.7	<0.0001
Hypertension, n (%)	65 (65.0)	91 (80.5)	0.0106
Diabetes, n (%)	7 (7.0)	5 (4.4)	0.4159
Chronic AF, n (%)	1 (1.0)	5 (4.4)	0.2183
History of CAD, n (%)	6 (6.0)	6 (5.3)	0.8274
History of CVD, n (%)	8 (8.0)	19 (16.8)	0.0537
History of open-heart surgery, n (%)	8 (8.0)	0	0.0020
Creatinine on admission (mg/dL)	1.2 ± 1.1.	1.2 ± 1.4	0.9955
Hemodialysis, n (%)	1 (1.0)	4 (3.5)	0.3738
Chronic lung disease, n (%)	14 (14.0)	1 (0.9)	0.0002
Marfan syndrome, n (%)	1 (1.0)	0	0.4695
Initial symptom of chest/back pain, n (%)	73 (73.0)	68 (60.2)	0.0483
Shock, n (%)	19 (19.0)	34 (30.1)	0.0617
Tamponade, n (%)	13 (13.0)	22 (19.5)	0.2035
Cardiopulmonary arrest before admission, n (%)	3 (3.0)	4 (3.5)	1.0000
AR ≥ moderate, n (%)	9 (9.0)	6 (5.3)	0.2935
Acute neurological complication, n (%)	10 (10.0)	13 (11.5)	0.7240
Malperfusion syndrome, n (%)	4 (4.0)	10 (8.9)	0.1540
Patients referred, n (%)	70 (70.0)	84 (74.3)	0.4803
Diagnosed at our hospital, n (%)	60 (60.0)	71 (62.8)	0.6716
Time from admission to surgery, min	197.0 ± 167.9	183.1 ± 158.6	0.5360
Time from onset to surgery, min	425.1 ± 257.1 (n=97)	595.8 ± 768.7 (n=100)	0.0391
Onset (am)	45 (45.0) (n=97)	60 (53.1) (n=100)	0.0101
Season, n (%)			0.1144
Spring	26 (26.0)	25 (22.1)	
Summer	21 (21.0)	12 (10.6)	
Fall	24 (24.0)	37 (32.7)	
Winter	29 (29.0)	39 (34.5)	
IMH, n (%)	13 (13.0)	22 (19.5)	0.363
Neck vessel dissection, n (%)	34 (34.0)	38 (33.6)	0.9449
Innominate dissection, n (%)	39 (50.0)	41 (47.7)	0.766
RCC dissection, n (%)	26 (33.3)	29 (33.7)	0.9581
RC dissection, n (%)	8 (10.3)	7 (8.1)	0.6386
LCC dissection, n (%)	21 (26.9)	22 (25.6)	0.8453
LC dissection, n (%)	15 (19.2)	6 (7.0)	0.019
Dissection extent, n (%)			0.0045
Ascending	3 (3.9)	4 (4.7)	
Arch	6 (7.7)	15 (17.4)	
Descending	7 (9.0)	20 (23.3)	
Abdominal	21 (26.9)	22 (25.6)	
Iliac	41 (52.6)	23 (26.7)	
Unknown	0	2 (2.3)	
Entry, n (%)			0.0018
Ascending	43 (43.0)	76 (67.3)	
Arch	36 (36.0)	28 (24.8)	
Descending	11 (11.0)	3 (2.7)	
Unknown	10 (10.0)	6 (5.3)	

**Table 2:** Intraoperative data Values are n (%) or mean ± standard deviation. AAR, ascending aortic replacement; CABG, coronary artery bypass grafting; AVR, aortic valve replacement; MAP, mitral annuloplasty; CPB, cardiopulmonary bypass; CA, circulation arrest; ACP, antegrade cerebral perfusion; RBC, packed red blood cells; FFP, fresh frozen plasma.

	Total cohort (n=213)		
Admission variables	Males (n=100)	Females (n=113)	p-value
AAR or hemiarch replacement	55 (55.0)	83 (73.5)	0.0049
Partial arch replacement	2 (2.0)	4 (3.5)	0.6866
Total arch replacement	43 (43.0)	26 (23.0)	0.0019
Artery cannulation site			
Axillary artery	3 (3.0)	8 (7.1)	0.1794
Femoral artery	63 (63.0)	76 (67.3)	0.5150
Axillary and femoral artery	34 (34.0)	27 (23.9)	0.1035
Ascending aorta	1 (1.0)	0	0.4695
Entry closure	80 (79.2)	105 (92.1)	0.0065
Frozen elephant trunk	22 (22.0)	10 (8.9)	0.0073
Aortic root plasty or replacement	13 (13.0)	3 (2.7)	0.0043
Concomitant procedures	12 (12.0)	20 (17.7)	0.2453
CABG	7 (7.0)	13 (11.5)	0.2607
AVR	1 (1.0)	0	0.4695
Peripheral bypass	3 (3.0)	3 (2.7)	1.0000
Others	2 (2.0)	7 (6.2)	0.1778
Operative time	447.4 ± 157.6	398.4 ± 122.1	0.0114
CPB time (min)	220.7 ± 71.7	194.9 ± 54.9	0.0033
CA time	41.6 ± 15.1	39.6 ± 8.9	0.2247
ACP, n (%)	68 (68.0)	77 (68.1)	0.9824
ACP time	50.0 ± 46.9	37.7 ± 38.7	0.0366
Rectal minimum temperature	22.7 ± 2.9	23.0 ± 2.7	0.4414
Intraoperative RBC units	9.2 ± 11.5	9.4 ± 6.9	0.8604
Intraoperative FFP units	17.9 ± 13.1	17.2 ± 12.5	0.7100
Intraoperative platelets units	16.3 ± 10.9	15.0 ± 9.7	0.3954

## Results

Preoperative data

**Figure 1:**
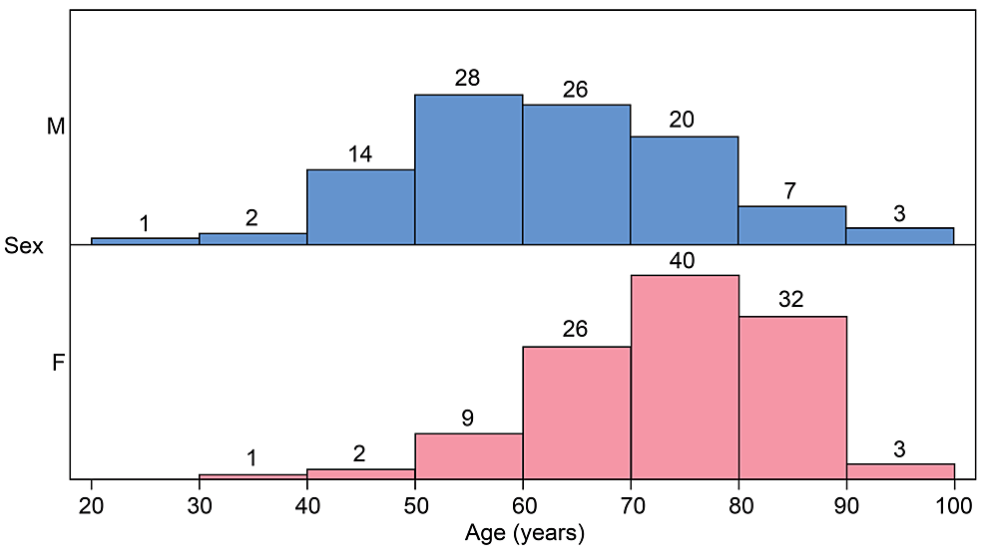
Relationship between age and sex in acute type A aortic dissection M: males, F: females.

Of the 213 patients with ATAAD who were included in the study, 100 (46.9%) were males and 113 (53.1%) were females. Female patients were significantly older than male patients (62.5 ± 13.4 vs. 79.2 ± 10.7 years; p<0.001) (Figure 1). Hypertension was significantly more common in females, whereas chronic respiratory failure and a history of cardiac surgery were more frequently observed in males. Although no significant difference was observed, females were more likely to present with shock than males (19.0% vs. 30.1%, p=0.0617). Females were less likely to develop ATAAD in the summer than males (21.0% vs. 10.6%, p=0.1144). Limited data showed that the onset of symptoms before noon was more common in females than in males (45.0% vs. 53.1%, p=0.0101). Females had significantly less thoracic back pain at onset than males (73% vs. 60.2%, p=0.0483). They also had a significantly longer time from disease onset to surgery than males (p=0.0391). However, the transfer rate did not differ between males and females.

Imaging findings

Approximately 80% of males had dissections extending to the abdominal aorta, whereas half of females had dissections remaining in the thoracic aorta (p=0.0045). Although no difference was noticed between dissection of the brachiocephalic, right common carotid, right subclavian, and left common carotid arteries, the left subclavian artery was more frequently dissected in males (19.2% vs. 7.0%, p=0.019). Additionally, no significant difference was observed in false lumen thrombosis between the two groups (13.0% vs. 19.5%, p=0.363). The primary entry point in the ascending aorta was more common in females (43.0% vs 67.3%; p=0.0018).

Intraoperative data

Significant differences in aortic procedures were observed between the sexes. Females underwent ascending aortic replacement more often (55.0% vs. 73.5%, p=0.0049), whereas total arch replacement was more frequently performed in males (43.0% vs. 23.0%, p=0.0019). The frozen elephant trunk technique was used more frequently in males than in females (22.0% vs. 8.9%, p=0.0073). Furthermore, males were more likely to undergo aortic root interventions than females (13.0% vs. 2.7%, p=0.0043). Surgery and cardiopulmonary bypass times were significantly longer in male patients, although the circulatory arrest time was similar in both sexes. Entry closure was more common in females than in males (79.2% vs. 92.1%, p=0.0065), and there were no differences in the number of intraoperative blood transfusions (Table 2).

Postoperative data

**Figure 2:**
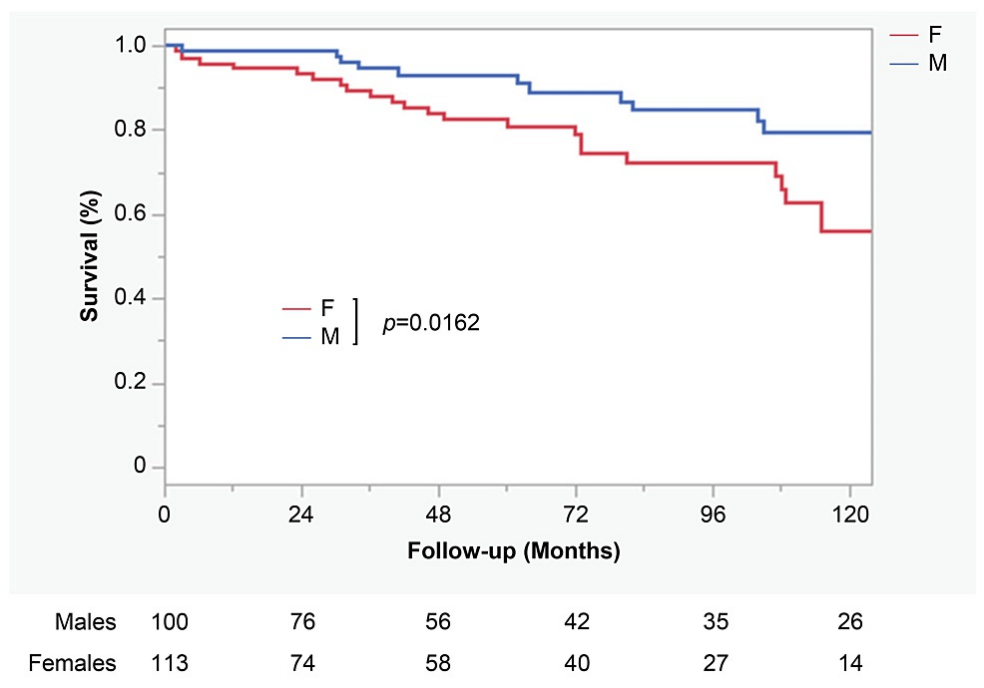
Unadjusted cohort long-term survival rate for all patients with log-rank test comparing males with females M: males, F: females.

No significant difference was observed in in-hospital mortality rates between sexes (9.0% vs. 10.6%, p=0.6923). The incidences of neurological complications and sepsis were similar in females and males; however, malperfusion was more common in females (3.0% vs. 8.9%, p=0.0751). As seen in Table 3, males had shorter ICU lengths of stay (6.0 ± 6.3 vs. 8.8 ± 11.3 days, p=0.0299). In the logistic regression analysis, the risk factors for in-hospital mortality were age (odds ratio (OR), 1.10; 95% confidence interval (CI), 1.02-1.10; p=0.0157), preoperative neurological complications (OR, 7.74; 95% CI, 1.28-46.6; p=0.0255), malperfusion syndrome (OR, 12.0; 95% CI, 1.96-73.8; p=0.0072), and operation time (OR, 1.01; 95% CI, 1.00-1.02; p=0.0002). Male sex did not influence in-hospital mortality (OR, 0.96; 95% CI, 0.18-5.21; p=0.9645). Risk factors specific to males were preoperative neurological complications (OR, 12.5; 95% CI, 1.23-122.1; p=0.0302) and operative time (OR, 1.01; 95% CI, 1.01-1.02; p=0.0003). As highlighted in Table 4, a risk factor specific to females was malperfusion syndrome (OR, 32.9; 95% CI, 6.46-163.4; p<0.0001). The unadjusted long-term survival was significantly higher in males than in females (p=0.0162). At 10 years, males had significantly better long-term survival rates in the unadjusted cohort (79.4% vs. 55.9%, p=0.02) (Figure 2).

**Table 3:** Postoperative data Values are n (%) or mean ± standard deviation. CVD, cerebrovascular disease; ICU, intensive care unit.

	Total cohort (n=213)		
Admission variables	Males (n=100)	Females (n=113)	p-value
Reoperation for bleeding	6 (6.0)	5 (4.4)	0.6042
New-onset CVD	10 (10.0)	15 (13.3)	0.4587
Reintubation	8 (8.0)	12 (10.6)	0.5130
Tracheostomy	8 (8.0)	17 (15.0)	0.1109
Requiring transient dialysis	10 (10.1)	10 (9.2)	0.8209
Requiring permanent dialysis	0	2 (1.8)	0.4987
Sepsis	4 (4.0)	4 (3.5)	1.0000
Malperfusion	3 (3.0)	10 (8.9)	0.0751
ICU stay, d	6.0 ± 6.3	8.8 ± 11.3	0.0299
Ventilator >3 d	43 (43.0)	48 (42.5)	0.9387
Hospital stay, d	35.8 ± 40.5	40.0 ± 60.3	0.5587
30-day mortality	7 (7.0)	6 (5.3)	0.6071
In-hospital mortality	9 (9.0)	12 (10.6)	0.6923

**Table 4:** Logistic regression of in-hospital mortality

	Multivariable analysis		
Admission variables	Odds ratio	95% Confidence interval	p-value
For all patients			
Age	1.10	1.02-1.1	0.0157
Preoperative acute neurological complication	7.74	1.28-46.6	0.0255
Malperfusion syndrome	12.0	1.96-73.8	0.0072
Operation time	1.01	1.00-1.02	0.0002
Male	0.96	0.18-5.21	0.9645
For males			
Preoperative acute neurological complication	12.5	1.23-122.1	0.0302
Operative time	1.01	1.01-1.02	0.0003
For females			
Malperfusion syndrome	32.9	6.46-163.4	<0.0001

## Discussion

Our findings revealed no significant differences in hospital mortality based on sex when comparing perioperative characteristics and surgical outcomes between females and males undergoing open aortic repair for ATAAD. However, females had significantly lower long-term survival rates than males in the unadjusted cohort. In addition, this study highlighted several important differences between females and males undergoing ATAAD repair; specifically, females were older at the onset of ATAAD and presented with fewer DeBakey type I dissections. They also had a higher incidence of onset before noon, presented more non-specific symptoms, and had a longer duration from onset to surgery. Furthermore, preoperative shock and malperfusion syndrome were more common in females than in males. Overall, female patients underwent more ascending replacements and fewer aortic root replacements.

In the absence of surgical intervention, ATAAD increases mortality by 1%−2%/hour by the second day after onset [12]. Therefore, early diagnosis and treatment should be performed to improve outcomes [17]. Our study revealed that females have a significantly longer time to intervention from onset than males. The reason for this prolonged intervention duration in female patients remains unclear. One plausible explanation could be the difference in the initial symptoms between males and females. Although few studies have focused on differences in symptoms, Nienaber et al. reported that females exhibited nonspecific early symptoms of ATAAD compared to males [18]. Consistent with their findings, our study showed that symptoms other than chest and back pain were significantly more common in females, thus potentially contributing to delayed diagnosis. Emergency and primary care physicians should be aware that approximately 40% of females with ATAAD present with nonspecific symptoms. Furthermore, many offer a rational explanation for the sex difference in onset time. In our study, females were more likely than males to develop ATAAD before noon. Although we did not investigate the specific hours of onset, it is widely acknowledged that clinical outcomes tend to deteriorate during nighttime hours due to a lack of inexperienced staff, or alternatively, hospital access may be adversely affected at night [19,20]. These sex-specific differences can result in prolonged intervention times and a poor preoperative status for female patients, since ongoing ATAAD-induced hemodynamic instability progressively worsens and causes serious complications, such as shock and malperfusion [9]. Our study also identified malperfusion syndrome as a risk factor of in-hospital mortality specific to females. Therefore, efforts aimed to shorten the time until intervention may improve survival rates in females [9,18].

Our findings revealed differences in the location of the primary entry site of ATAAD between males and females, with approximately two-thirds of females having the primary entry site in the ascending aorta. Furthermore, DeBakey type I was more common in males, whereas type II was more common in females. These differences led to increased female ascending aorta replacements using our entry-oriented strategy. Aortic root interventions were performed more frequently in males than in women, which is consistent with the results of a previous study [5-11]. However, such root interventions increase operation and cardiopulmonary bypass times, which can lead to increased mortality [21-23]. In our study, an increased operative time was a male-specific risk factor for early mortality. Current guidelines [24] dictate that aortic interventions are determined by the diameter of the aorta regardless of sex, which may result in disproportionately excessive interventions in males. It would seem favorable to focus more on sex-specific normal aortic diameters [5] and we should make a decision after careful consideration in adopting extensive aortic surgery for males with ATAAD.

Surgical mortality did not differ significantly between the sexes (9.0% vs. 10.6%, p=0.6923). However, our study demonstrated that females had lower long-term survival rates than males in the unadjusted cohort, which is inconsistent with previous reports [6,10,11]. This difference can be attributed to the age of the patient population. Unlike many other studies, our study included many elderly patients, and the multivariate analysis identified age as a risk factor for in-hospital mortality. Prior reports have shown that females develop aortic dissection at an older age than males because of the progression of arteriosclerosis caused by decreased estrogen levels [25,26]. Given this fact, it seems natural that the long-term survival rates were lower in females than in males. Previous studies have shown that surgery can be performed safely in elderly and younger patients with ATAAD [27,28]. Therefore, even though females with ATAAD may be older and exhibit poorer preoperative conditions than males, treatment strategies should not be based on sex considerations.

Limitations

This study had some limitations. Firstly, this study was conducted at a single center in Japan. The findings of this study cannot be generalized because the expertise of surgeons, the number of people, and the volume of procedures vary depending on the facility. In addition, our study included only patients who survived surgery and excluded those who died before the procedure. This exclusion criterion might introduce a potential bias in our analysis due to an underrepresentation of cases with more severe conditions or higher mortality risks. Lastly, the small sample size may have been insufficient to detect subtle differences.

## Conclusions

Several differences were found between females and males who underwent ATAAD repair. Females had more nonspecific symptoms and a longer intervention time than males. Notably, malperfusion syndrome emerged as a specific risk factor for in-hospital mortality in females; thus, shorter interventions are recommended for female patients. Conversely, males underwent more extensive surgeries than females, and prolonged operative time was a risk factor. Caution should be exercised when performing extensive surgeries on male patients. Understanding the sex differences in medical treatments may lead to improved outcomes. Future studies should involve a larger sample size and consider using a large dataset from the whole of Japan.
